# Use of Human Senses as Sensors

**DOI:** 10.3390/s90503184

**Published:** 2009-04-27

**Authors:** Yoshiaki Sugawara, Chie Sugimoto, Sachiko Minabe, Yoshie Iura, Mai Okazaki, Natuki Nakagawa, Miwa Seto, Saki Maruyama, Miki Hirano, Ichiro Kitayama

**Affiliations:** 1 Department of Health Science, Prefectural University of Hiroshima, Hiroshima 734-8558, Japan; 2 Division of Titanium Oxide Products, Ohno Sekiyu Co. Ltd., Hiroshima 730-0005, Japan; E-Mail: kitayama.i@ohno-group.co.jp (I.K.)

**Keywords:** Human senses, sensory evaluation, photocatalytic efficacy of TiO_2_, potency of essential oils

## Abstract

This paper is an overview of our recent findings obtained by the use of human senses as sensors, suggesting that human senses might be indispensable sensors, not only for practical uses but also for gaining a deeper understanding of humans. From this point of view, two kinds of studies, both based on semantic responses of participants, deserve emphasis. One study assessed the efficacy of the photocatalytic elimination of stains or bio-aerosols from an air environment using TiO_2_ as well as the photocatalytic deodorizing efficacy of a TiO_2_-type deodorizer; the other study evaluated the changes in perception of a given aroma while inhaling the fragrance of essential oils. In the latter study, we employed a sensory test for evaluating changes in perception of a given aroma. Sensory tests were conducted twice, when participants were undergoing the Kraepelin mental performance test (mental arithmetic) or an auditory task (listening to environmental natural sounds), once before the task (pre-task) and once after the task (post-task). The perception of fragrance was assessed by 13 contrasting pairs of adjectives as a function of the task assigned to participants. The obtained findings illustrate subtle nuances regarding how essential oils manifest their potency and how olfactory discrimination and responses occur in humans.

## 1. Introduction

Sensory evaluation is a method of measuring consciousness developed primarily in experimental psychology and mathematical psychology [1-5]. Sensory experiences can be reported using verbal (semantic) methods. To elucidate the psychological efficacy of essential oils, the authors have endeavored to develop a sensory (verbal) measure of the perceived odor quality for a given aroma and analyze it statistically [6-14]. Given the hypothesis that human senses might be indispensable sensors not only for practical uses but also for gaining a deeper understanding of humans, the present research is an overview of our past findings of the research achievements of essential oils over the decade as well as the recent achievements for examining the efficacy of the photocatalytic elimination of stains or bio-aerosols from an air environment using TiO_2_ and the deodorizing efficacy of a TiO_2_-type deodorizer.

Since Matsunaga and coworkers reported in 1985 the photocatalytic sterilization of microbial cells by titanium oxide (TiO_2_) upon illumination [15], it has been known that TiO_2_ possesses many functions, not only for photocatalytic sterilization of microbial cells, but also for use in photocatalytic decomposition of organic compounds, photocatalytic removal of stains and molds from indoor environments, and photocatalytic elimination of bio-aerosols from air environments [16-23]. Many groups have used this novel technology for a variety of medical applications and for incorporation into industrial goods. In the first study, semantic responses from participants were used to determine the efficacy of photocatalytic elimination of stains or bio-aerosols from air environments by TiO_2_. For this purpose, one of the sliding doors of a storeroom placed in open air was coated with TiO_2_ emulsion and left for two years; the other door was not coated. Participants' impression changes for the sliding doors were assessed by inquiry on an 11-point scale (-5 to +5) using 13 contrasting pairs of adjectives. The panels in our study should be first described. We employed untrained (unexperienced) individuals as panelists, even though our sensory test can be categorized as a group of descriptive sensory analysis with a prerequisite of use of trained individuals as panelists [24-27]. Ours follows the method that was developed for evaluation of the perceived odor quality of essential oils under our original premise of usage of untrained individuals as panelists [6-14]. Accordingly, assessment by inquiry was conducted twice: once for the TiO_2_ coated door and once for the uncoated door. The mean of impression difference between the score of the first inquiry for the coated door and the second inquiry for the uncoated door was plotted against the descriptors. The obtained bar graph (sensory evaluation spectrum) showed an upward tendency with a positive value against the descriptors. This shows that the coated door was superior to the uncoated one in terms of the setting semantic variables. It is suggested that the sensory spectrum obtained may provide information for use in the assessment of diverse sorts of functions of TiO_2._

In the second study, we focused on the photocatalytic efficacy of a TiO_2_-type deodorizer based on the semantic responses of the participants. Statistical analysis of this measure was conducted at the same time. The deodorizer used in this study was commercially available, and sensory tests were conducted after setting the deodorant in participants' own home refrigerators using as indices the impression of the smell within the refrigerator.

The final and the most recent study reported our findings obtained by verbal (semantic) and nonverbal responses to odorants by participants. Many reports have been published in the literature concerning odors used to alter mood, alertness, and sexual arousal [28-30]. Perfumes, room fragrances and incense have been used for self-adornment and modification of the living environment since ancient times. The treatment practice known as Aromatherapy, started in France in the early 20th century, has been developing rapidly. Essential oils are now extensively utilized in the context of Aromatherapy and Aroma Wellness. In spite of these natural uses of odors and the empirical/anecdotal accounts of their effects, little is known of their scientific basis [31]. We used a sensory measure to explore the possible psychological effects on humans of inhaling essential oils. Sensory tests by inquiry were also employed and conducted twice, when participants were undergoing the Kraepelin mental performance test (mental arithmetic) or an auditory task (listening to natural environmental sounds), once before the task (pre-task) and once after the task (post-task) [6-8]. Thirteen pairs of adjectives were used similarly as in the first study, and each was scored on an 11-point scale. As a function of the type of task assigned to the subjects, aroma perception was evaluated in terms of the change of the scores in the questionnaires. The mean perception differences before and after each type of task were then depicted against the descriptors as a sensory evaluation spectrum. These kinds of tests have been previously conducted, and the sensory features of 21 essential oils and one monoterpenoid (linalool) have been detailed. The sensory features obtained indicate that inhalation of odorants may result in different subjective perceptions depending on the type of task assigned to participants. To identify further possible physiological changes while inhaling odorants, fingertip skin temperature changes were monitored using a multi-channel skin thermometer [13,14]. The results obtained from participants' verbal (semantic) and nonverbal responses to odorants illustrate subtle nuances regarding how essential oils manifest their potency and how olfactory discrimination and responses occur in humans [14].

## 2. Sensory evaluation Study Regarding the Efficacy of Photocatalytic Elimination of Stains or Bio-Aerosols from Air Environments by TiO_2_

**Figure 1.**
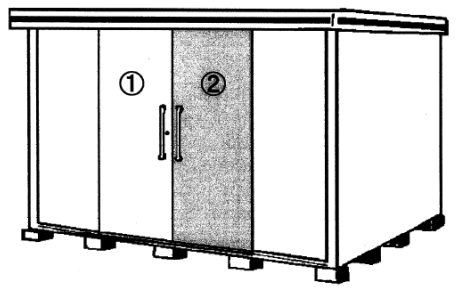
A picture of the storeroom constructed as an experimental plant in this study. One of the sliding doors (designated as number 2) of the storeroom placed in the open air was coated with a TiO_2_ emulsion and left for two years, while the other door (designated as number 1) was not coated.

In this study, semantic responses from participants were used to determine the efficacy of photocatalytic elimination of stains or bio-aerosols from air environments by TiO_2_. For this objective, (as shown in Figure 1) one of the sliding doors (size: 72 cm × 176 cm) of a storeroom (width: 300 cm; breadth: 90 cm; height: 195 cm) which had been placed in the open air was coated with a TiO_2_ emulsion (Miracle Titan M-2; anatase type; average diameter = 15 nm) and left for two years, while the other door was uncoated and used as a control. Using a sprayer, a TiO_2_ emulsion (density: 0.8 weight %) was sprayed on the wall of the sliding door, and a TiO_2_ film was then formed after drying with a thickness of about 1 μm.

We screened five judges who, through debate, chose 23 contrasting pairs of adjectives (Table 1), assessing the efficacy of the photocatalytic elimination of stains or bio-aerosols from air environments using TiO_2_. An additional five panelists were requested to stand in front of the storeroom and mark the applicable threshold values (scores) for every adjective pair listed in Table 1, including 0 – “unfavorable or unsuitable,” 1 – “preferable to choose” and 2 – “favorable or suitable.” The following were deemed by the judges to be appropriate, that is, all judges marked them at more than level 1: bright – dark, clear – heavy, pure – sandy, clean – dirty, agreeable – disagreeable, comfortable – uncomfortable, likeable – dislikable, fresh – stale, fine – dusty, transparent – opaque, brilliant – stringy, glossy – gloomy, and slippery – coarse.

Using the 13 contrasting pairs of adjectives, sensory tests by inquiry were conducted twice, once for the TiO_2_ coated door and again for the uncoated door. An 11-grade evaluation was made from +5 to -5, with 0 as the middle score and without any symbolic representation of the numbers in a similar way to the aforementioned applicable threshold values or the Linkert scale [32]. Fifty-two panelists completed the study as participants. Among these, 18 were nonstudent volunteers with ages ranging from the 20s to 70s; they formed panel A (nonstudent panel). The other 34 were all female students, ages 19 and 20, attending the Prefectural University of Hiroshima. They formed panel B (student panel). No participant overlapped as a panelist in these inquiries.

**Figure 2.**
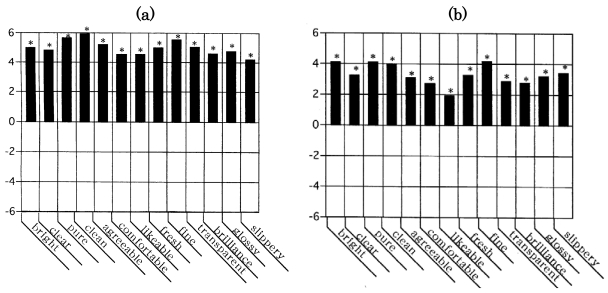
The sensory evaluation spectra: the resulting impression changes of the subjects between the TiO_2_ coated door and the uncoated door. Sensory evaluation spectrum obtained from nonstudent panel (*n* = 18), (b) Spectrum from student panel (*n* = 34). The assessment by inquiry was conducted two times: one was for the TiO_2_ coated door and the other was for the uncoated door. The resulting impression changes of the subjects between the TiO_2_ coated door and the uncoated door are depicted against the 13 impression descriptors.

The mean of the impression difference between the score of the first inquiry for the coated door and the second inquiry for the uncoated one was plotted against the descriptors. The obtained bar graph (sensory evaluation spectrum) is represented in Figure 2. Figure 2a indicates the results obtained from panel A, while Figure 2b shows the results from panel B. Both figures demonstrate an upward tendency with a positive value in terms of the 13 descriptors. This suggests that the coated door was superior to the uncoated one from the viewpoints of “bright,” “clear,” “clean,” “likeable,” “fine,” “brilliant,” and the other setting descriptors. The statistical significance of the change of score between the first inquiry and the second inquiry was evaluated by Student's *t*-test. The obtained statistical significance was indicated on every descriptor as follows: marked with an asterisk (*) if the impression difference was significant with *p* < 0.05 and unmarked if *p* ≥ 0.05. As shown in Figure 2, the 13 descriptors were all regarded to be significant in both spectra, as all items were marked (*) in both. If there was an effective and positive correlation between the impression and the installation of TiO_2_, the descriptors regarded as significant by the *t*-test have a positive value and are shown above the horizontal axis. On the other hand, negative values appear below the axis if there was an ineffective or negative correlation between the impression and the installation of TiO_2_. Both spectra indicate a striking resemblance so that the improvement obtained by the installation of TiO_2_ was demonstrated statistically. TiO_2_ has been shown to possess multiple functions. These sensory spectra may contribute to the assessment of additional diverse functions of TiO_2_ through the use of semantic evaluation.

## 3. Sensory Evaluation of the Efficacy of A Photocatalytic Deodorizer

Given the hypothesis that sensory spectra might assist the assessment of functions of TiO_2_, we studied the photocatalytic efficacy of a TiO_2_-type deodorizer based on the perceptional changes of participants evaluating refrigerator odors. The deodorizer used for this study was a commercially available one with a surface layer coated with TiO_2_ powder through a reaction process in which aqueous colloid solution can be dried and calcined in a stove. This was purchased from Ohno Sekiyu Co. Ltd. (Hiroshima, Japan). The length, width and height of the deodorizer were 9, 8 and 0.5 cm, respectively, Sensory tests were conducted when the deodorant placed in the participants' own home refrigerators using as indices the impressions of the smell within the refrigerator. The impression items composed of 13 contrasting adjective pairs were selected by the 5 judges from a list of 25 level pairs similar to the aforementioned process: fresh – stale, refreshing – not refreshing, clear cut – sluggish, clear – opaque, clean – dirty, natural – artificial, calm – irritating, harmonious – inharmonious, agreeable – disagreeable, comfortable – uncomfortable, pure – musty, refined – vulgar, and pleasant – unpleasant. For each of these impression items, an 11-point evaluation from +5 to −5 was made. To ascertain this measure, a repeatability test was conducted comparing the studies undertaken in 2003 and 2004. Fifty-six panelists completed the study as participants. They were female students attending the Hiroshima Prefectural Women's University (renamed Hiroshima Prefectural University on April 1, 2005) with ages ranging from 19 to 22. Among these, 21 participated in the study that was carried out in 2003; this group formed panel C. The other 35 engaged in the work done in 2004 and formed panel D. No participant was a panelist in both studies.

When setting the deodorant in the participants' own home refrigerators, a series of sensory tests were conducted using as an index the impression of refrigerator odors. The aforementioned 13 contrasting adjective pairs were used for the sensory evaluation, and each was scored on an 11-point scale (-5 to +5). The assessment by inquiry was conducted three times: once immediately before placing the deodorant in the refrigerator, again after keeping it in the refrigerator for a week, and one week after removing it from the refrigerator.

**Figure 3.**
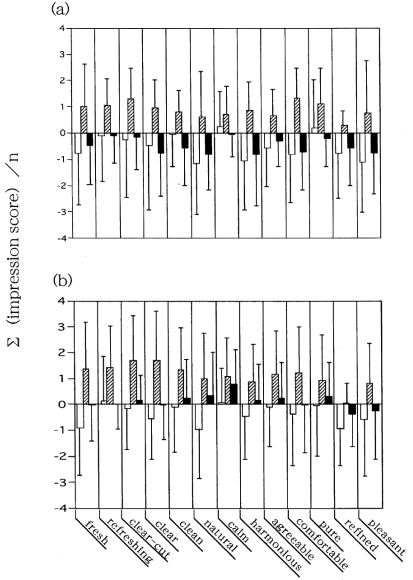
The mean ± standard deviation profile obtained by sensory test using as an index the change of impression of refrigerator odors. (a) The study undertaken in 2003 (*n* = 21), (b) The study in 2004 (*n* = 35). The assessment by inquiry was conducted three times; once immediately before placing the deodorizer in the refrigerator, again after keeping it in the refrigerator for one week, and finally one week after removing it from the refrigerator. The resulting perceptional changes of the subjects related to refrigerator odors assessed by 13 contrasting pairs of adjectives on an 11-point scale (-5 to +5) are depicted. Open bars represent the first inquiry before installation of the deodorizer, diagonal shaded bars represent the second inquiry after installation of the deodorizer, and filled bars represent the third inquiry following removal of the deodorizer.

Figure 3 shows a mean-deviation profile for the sensory test. The following focuses on results obtained from panel C (Figure 3a). The negative values of the scores for the first inquiry (i.e., the inquiry before installation of the deodorizer) appear below the horizontal axis, while the scores for the second (i.e., the inquiry after installation of the deodorizer) have risen to a positive value. This tendency reverses for the third (i.e., the inquiry after removal of the deodorizer) with a negative value shown below the axis. In each inquiry, however, a large standard deviation value for each descriptor was remarkable against a smaller value of the mean, although a decreasing tendency of the standard deviation was observed in the second inquiry as compared with the first and third. No discrepancy was observed in the study obtained from panel D (Figure 3b). These were statistically non-significant, considering the large standard deviation.

**Figure 4.**
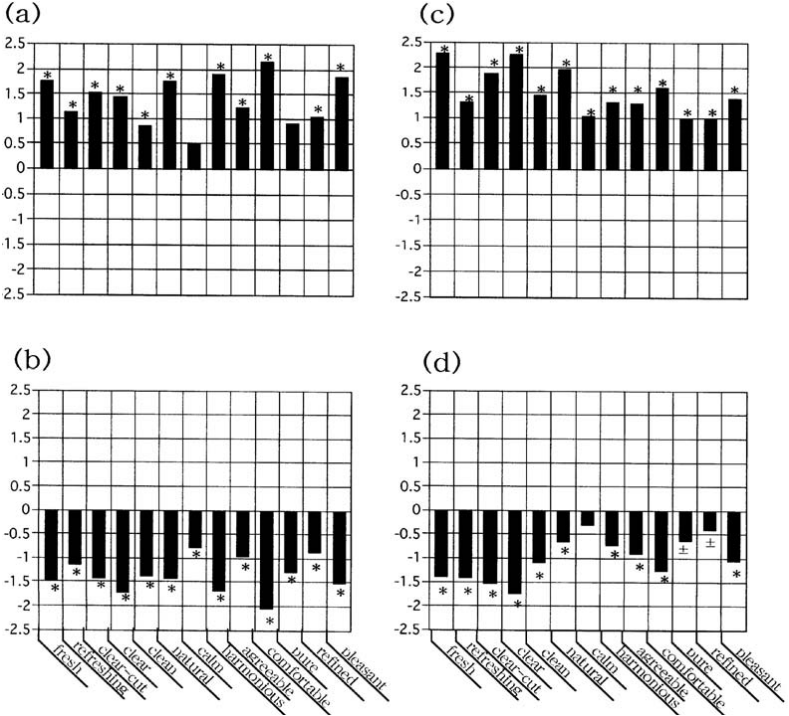
Sensory evaluation spectrum for the efficacy of a photocatalytic deodorizer. (a) and (b): The study undertaken in 2003 (*n* = 21), (c) and (d): The study in 2004 (*n* = 35). Sensory spectra of (a) and (c) were obtained when the deodorizer was placed in the refrigerator, while those of (b) and (d) were acquired after its removal. The statistical significance for each descriptor was tested by Student's *t*-test, and the descriptor regarded to be significant at a probability value of *p* < 0.05 is indicated with a single asterisk (*).

The scores recorded in the second inquiry were subtracted from the respective values obtained in the first (the second minus the first). Similarly, the scores registered in the third were subtracted from the respective values in the second (the third minus the second). Figure 4 depicts the resulting sensory evaluation spectrum. Figure 4a as well as Figure 4c show a spectrum indicating the impression difference between before and after installation of the deodorizer, while Figure 4b, as well as Figure 4d, gives the impression difference between after installation of the deodorizer and removal of the deodorizer. These are represented by a bar graph, and the statistical change for each descriptor was assessed by the *t*-test, in which the statistical significance is marked with an asterisk (*) if the impression difference was significant at a probability value of *p* < 0.05. Figures 4a and b were obtained from panel C, while Figures 4c and d were from panel D.

In a sensory evaluation spectrum, if there is a positive correlation between the change of smell in the refrigerator and the installation of the deodorizer the descriptors regarded as significant by the *t*-test have a positive value and are shown above the horizontal axis; negative values appear below the axis if there is a negative correlation between the change of the smell and the installation of the deodorizer or removal of the deodorizer. It can be seen in Figure 4 that both spectra a and c show odor improvement when the deodorizer was placed in the refrigerator. However, the sensory features of spectra b and d are both perfectly reversed to the respective spectra of a and c, indicating a worsening smell once the deodorizer was removed from the refrigerator.

A question might be raised as to whether the obtained sensory profile can be regarded as statistically significant as a whole of the change of the spectrograph. We dealt with this issue by applying the sign test with *n* = 13, since 13 pairs of descriptors were used in our sensory test. It should be noted that the obtained sensory spectrum reached significance (*p* < 0.05) if the number of the descriptors regarded as significant at a probability value of *p* < 0.05 by the *t*-test were > 10 items among 13 descriptors. Meanwhile if this value was < 3, a null hypothesis could be rejected.

This value can be calculated from Figure 4 as 11 for spectrum a, 13 for b, 13 for c and 10 for d, so that the change of the obtained spectra was significant (*p* < 0.05) without d. In spectrum d, the change was not significant statistically, although 10 items were regarded as significant (*p* < 0.05) by the *t*-test among 13 descriptors. If we changed the level of significance from *p* < 0.05 to *p* < 0.1, two descriptors, pure – musty, and refined – vulgar, would be significant (0.05 ≤ *p* < 0.1). If these are marked as ± in the graph (Figure 4d) and one considers the two ± would change to one +, the change of spectrum d would be significant as a whole of the change of the spectrograph on the basis of the sign test (*p* < 0.05).

The statistical significance of each impression descriptor was marked and scored as follows: (1) * and significance score = 1, if the impression difference was regarded to be significant with *p* < 0.05, (2) ± and significance score = 0.5, if regarded to be significant with *p* ≤ 0.05 - 0.1, and (3) unmarked and significance score = 0, if regarded to be insignificant with *p* ≥ 0.1. If the addition of these scores is defined as the following total significance score = Σ^13^_i = 1_ (significance score of descriptor)_i_, this value would serve as an index to judge whether the obtained sensory profile can be regarded as statistically significant as a whole of the change of the spectrograph. This value should be required to be > 10 if the obtained sensory spectrum is significant (*p* < 0.05). With a value of < 3, a null hypothesis could be rejected.

It was thus shown that a pair of sensory spectra could represent a functional aspect of the deodorizing efficacy of a photocatalytic deodorant: one was a spectrum for installation of deodorant in a refrigerator, and the other was for its removal. The former spectrum shows that odors were lessened by the use of the deodorizer, whereas the latter indicates that the odors were intensified by removing the deodorizer.

## 4. Human Verbal and Non-Verbal Responses to Odorants While Inhaling the Fragrances of Peppermint and Spearmint Essential Oils and Linalool [13,14]

In this section, we focus on our findings in previous papers that appeared in the *Flavour and Fragrance Journal* [13] and the *International Journal of Essential Oil Therapeutics* [14] in 2006 and 2008, respectively. In both we examined the relationship between mood changes and odor and its physiological effects by focusing on the possible verbal and non-verbal changes in humans induced by smelling the fragrances of peppermint and spearmint essential oils and linalool.

Essential oils used were products by Fleur (London, UK). Linalool was purchased from Kanto Kagaku Co. Ltd (Tokyo, Japan). To identify the optimal concentration of each for inhalation experiments, preliminary sensory tests were performed according to the specifications of Sugawara *et al.* [8]. Serially diluted solutions of 1/1, 1/10, 1/50, 1/100, 1/1,000 and 1/10,000 of a given aroma to diethyl phthalate were presented to several judges (usually five) via an inhalator composed of a glass inhalator device and a 300 mL flask with a ground-glass stopper. An inhalator flask was loaded by applying 200 μL of each diluted solution to a small strip of filter paper on the bottom, sealed with a ground-glass stopper and moistened with fragrance.

Regarding the intensity of fragrance within the inhalator flask, the following applicable odor detection threshold values (scores) were established: 0 – odorless, 1 – odor barely detectable and the nature of the odor cannot be ascertained, 2 – very weak odor but the nature of the odor can be discriminated, 3 – weak odor but the odor can be readily detected, 4 – strong odor, and 5 – odor so strong that it cannot be tolerated. Five judges were requested to mark from 0 to 5 each serially diluted test solution. In the experiments, dilutions of 1/10 for spearmint and linalool and 1/100 for peppermint were employed, as these concentrations were deemed by the judges to have a score of 3 and above.

Aroma perception was evaluated by the following 13 impression descriptors consisting of contrasting pairs of adjectives: fresh – stale, soothing – active, airy – heavy, plain – rich, natural – unnatural, elegant – unrefined, soft – strong, pleasant – unpleasant, warm – cool, comfortable – uncomfortable, woodsy – not woodsy, floral – peppery, lively – dull. We used the Uchida – Kraepelin test as a mental arithmetic task and listening to environmental (natural) sounds as an auditory task. The Uchida – Kraepelin test involves administering adjacent rows of numbers (100 numbers per row) to the subjects. Subjects perform simple additions using numbers within a row. Each row was worked on for 40 seconds before changing to the next row (5 min total). The auditory task (5 min total) was performed while sitting on a chair and listening to natural sounds on a compact disc player such as bird songs or the murmuring of a small stream.

**Figure 5.**
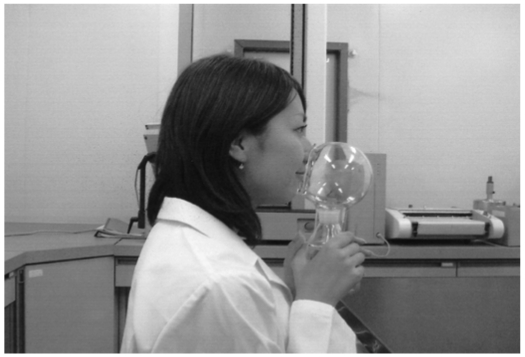
Picture of the sensory test when the subject inhaled the fragrance of a given aroma of essential oils.

A sensory test was conducted before and after the task, as presented in Figure 5. Thirteen impression descriptors were scored on an 11-point scale (-5 to +5). The pre-post task difference in the score of each of the impression descriptors was evaluated by the *t*-test. The statistical significance of each impression descriptor was marked and scored as follows: (1) * and significance score = 1, if the impression difference was regarded to be significant with *p* < 0.05, (2) ± and significance score = 0.5, if regarded to be significant with *p* ≤ 0.05 - 0.1, and (3) unmarked and significance score = 0, if regarded to be insignificant with *p* ≥ 0.1. The addition of these scores provided the following total significance score = Σ^13^_i = 1_ (significance score of descriptor)_i_.

**Figure 6.**
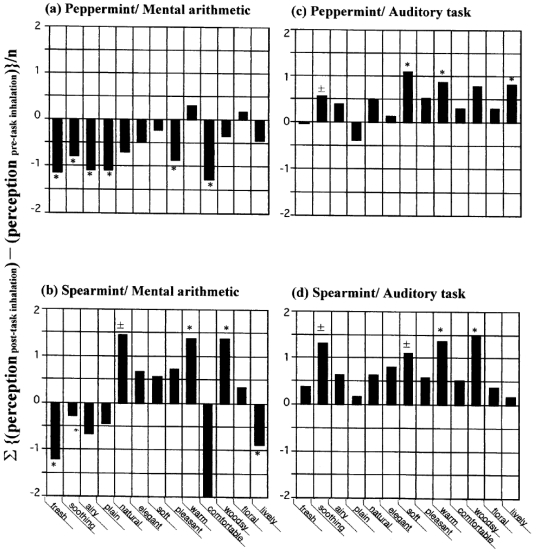
Task dependent sensory spectra for peppermint and spearmint essential oils. Redrawn from Sugawara *et al.* [14]. A sensory test was conducted twice before and after the task assigned to the subjects, in which aroma perception was evaluated by 13 impression descriptors consisting of contrasting pairs of adjectives. The pre-post task difference in the score of each of the impression descriptors is plotted on the ordinate as a bar graph. The statistical significance evaluated by the *t*-test of each descriptor was marked with a single asterisk (*) if the pre-post impression difference was regarded significant with *p* < 0.05, ± if regarded significant with *p* ≤ 0.05 - 0.1, and unmarked if *p* ≥ 0.1. The number of subjects were (a) 20, (b) 18, (c) 23, (d) 18.

These types of tests have been carried out for the past decade, and the sensory features of the following 21 essential oils and one monoterpenoid (linalool) have been detailed so far: basil, bergamot, cardamom, chamomile, cinnamon, clove, cypress, geranium, ho leaf/wood, juniper, lavender, lemon, lime, marjoram, orange, palmarosa, peppermint, rosemary, sandalwood, spearmint, and ylang ylang. The sensory features of peppermint and spearmint essential oils and linalool were most evidently task dependent. The sensory features related to peppermint and spearmint essential oils are shown in Figure 6.

If there was a favorable correlation between the fragrance of a given aroma and the type of task, the descriptors regarded as significant by the *t*-test have a positive value and are shown above the horizontal axis; negative values appear below the axis if there was an unfavorable correlation between the fragrance and the type of task. As shown in Figure 6a, in the peppermint spectrum during mental arithmetic, there was an unfavorable correlation between the fragrance and the type of task assigned to the subject. On the other hand, the spectrum of peppermint in Figure 6c was positive during the auditory task. Similar results were found for spearmint (Figures 6b and d). These findings suggest that inhalation of fragrant compounds might result in different subjective perceptions depending on the type of task assigned to the subjects. Similar task dependent effects were also observed with linalool, as shown later.

The value of a total significance score was calculated as 6.0 for peppermint and 4.5 for spearmint in relation to mental arithmetic, and 3.5 for peppermint and 3.0 for spearmint in relation to the auditory task. It should be noted that there were no statistically significant differences between total significance scores according to task. On the basis of the sign test with *n* = 13, this value should be required to be > 10, while a null hypothesis could be rejected if the value was < 3. All the cases shown in Figure 6 were insignificant, but these were employed with total significance scores of ≥ 3. Like peppermint and spearmint, similar values (≥ 3) were found for linalool, as shown later too.

**Figure 7.**
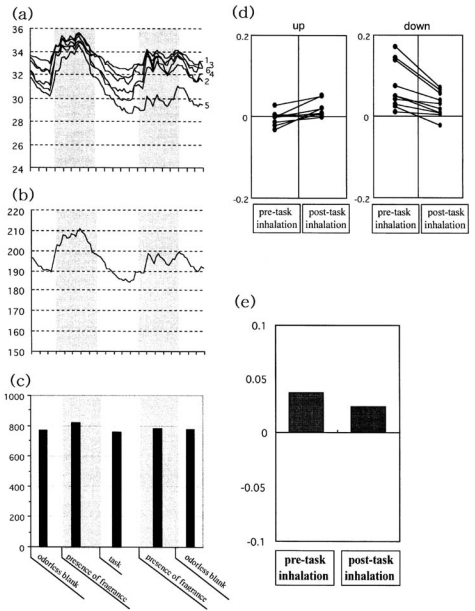
The observed skin temperature changes following inhalation of peppermint in relation to the auditory task. Redrawn from Sugawara *et al.* [14]. The numbers assigned to the graph represent the sensor spots on the left hand: 1, the tip of the thumb; 2, the tip of the first finger; 3, the tip of the second finger; 4, the tip of the third finger; 5, the tip of the fourth finger, and 6, the palm. The number of subjects was 20.

On the basis of the verbal responses elicited by smelling presented stimuli, possible skin temperature changes while inhaling the fragrance of two essential oils (peppermint and spearmint) and one monoterpenoid (linalool) were concurrently monitored by a multi-channel skin thermometer as a function of the task assigned to the subjects in order to examine the relationship between subjective emotional perception of odor and its physiological effects [13,14]. Figure 7 depicts an example of skin temperature experiments in which the subjects inhaled peppermint before and after an auditory task.

Skin temperature measurements were conducted in a climatic chamber at 20 °C and 60% relative humidity. The procedure was first explained to the subjects, then they were encouraged to relax and allowed to rest quietly for 5 min before beginning the test. Skin temperature was recorded using a multi-channel thermometer (Anritsu AM-7052) equipped with a data collector. Thin surface thermistors (2 × 10 mm) were used as recording sensors. They were attached with adhesive tape to the tips of every left finger and to the palm at the base of the first finger on the left hand. As shown in Figure 7a, every skin temperature curve recorded from the tip of each left finger and from the left palm showed a small but considerable fluctuation. The measured data were stored on a personal computer (Dell-OptiPlex Gn+EM) at a sampling rate of 15 s via an A/D converter connected on-line to the multi-channel thermometer. As shown in Figure 7b, this allowed summation of the data from each channel every 15 s so that a single-temperature curve could be obtained for all the measurement points.

**Figure 8.**
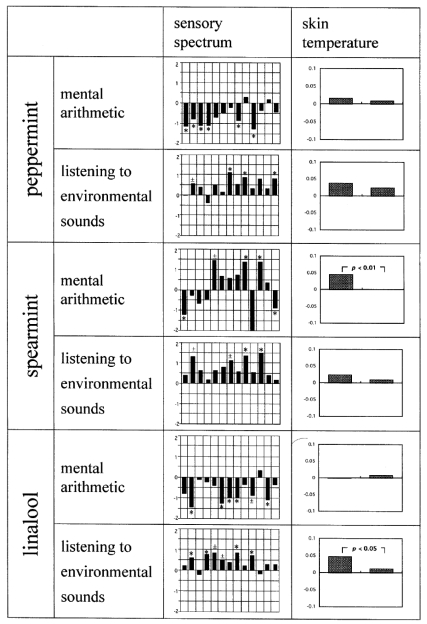
Summary of the obtained verbal and nonverbal responses to odorants following inhalation of peppermint and spearmint essential oils and linalool in terms of sensory evaluation spectrum and net intensity skin temperature changes, when observed as a function of extraneous condition assigned to the subjects. As for spearmint, the number of subjects in relation to nonverbal test was 17 for mental arithmetic and 18 for the auditory task (listening to environmental sounds). As for linalool, the number of subjects was 20 for mental arithmetic and 22 for the auditory task in relation to verbal test; 20 for mental arithmetic and 20 for the auditory task in relation to nonverbal test.

Individual variation was evident between each trial in an experimental run. In each trial the minute-based mean average temperature (Figure 7c) was then calculated according to the odor presentation periods given at the bottom of the graph, in which assessment of the changes in skin temperature was conducted via the specifications of Sugawara *et al.* [13]. On the basis of a minute-based intensity profile (bar chart) that was constructed by the integration of temperature curves between each section of the skin temperature measurement protocol (mean temperatures at minute-intervals), each experimental run net intensity change in skin temperature was calculated according to the following formula with respect to presentation of the odorless blank and the target fragrance: (T^MMA^_odor_ - T^MMA^_o_) / T^MMA^_o_, where T^MMA^_odor_ is the observed intensity of the minute-based mean average temperature during odor presentation, and T^MMA^_o_ is the respective intensity of the odorless blank. This equation produced the results depicted in Figures 7d and e. The values were calculated pre and post task in each trial. In each experimental run, the cases with upward (increasing) skin temperature change pre to post task are plotted on the left, while those with downward tendencies are represented in the right panel (Figure 7d). The summarized mean values of net intensity changes obtained from pre and post task inhalations are depicted as a bar graph (Figure 7e). Like peppermint, similar results were found for spearmint and linalool in terms of net intensity skin temperature changes. In terms of sensory evaluation spectrum and net intensity skin temperature changes, the obtained verbal and nonverbal responses to odorants following inhalation of peppermint and spearmint essential oils and linalool are summarized in Figure 8.

## 5. Discussion

As aforementioned, sensory analysis comprises a variety of tools or tests that can be used for subjective or objective evaluation of some sensory properties. Among these, our sensory test can be categorized as a group of descriptive sensory analysis. It is well known that descriptive sensory analysis consists of training a group of individuals (generally 6 to 12) to identify and quantify specific sensory attributes [24-27]. Unlike this, the present research used untrained (unexperienced) individuals as panelists.

It is sure that if one uses untrained individuals as panelists, factors such as interest for sensory target, sensitivity to stimuli, onset of fatigue and so on vary between panelists. As previously mentioned in the second study, our sensory tests were carried out after setting the deodorant in the participants' own home refrigerators using as indices the untrained participants' impression of the smell within the refrigerator. The questionnaire assessment was conducted tree times: once immediately before placing the deodorant in the refrigerator, again after keeping it in the refrigerator for a week, and one week after removing it from the refrigerator. The obtained mean ± standard deviation profile (Figure 3) demonstrated that a large standard deviation value was remarkable against a smaller value of the mean for the 13 descriptors. Regardless of such a large standard deviation value against a smaller value of the mean, it was shown that a pair of sensory spectra could represent a functional aspect of the deodorizing efficacy of a photocatalytic deodorant: one was a spectrum for installation of deodorant in a refrigerator, and the other was for its removal. The former spectrum shows that odors were lessened by the use of the deodorizer, whereas the latter indicates that the odors were intensified by removing the deodorizer. To ascertain this point, a duplicate sensory test was conducted. The obtained findings (Figure 4) showed a satisfactory result not only in reproducibility but also in consistency, in statistical significance and in accuracy, as an objectively measure for human responses to stimuli. These circumstances were identical in the first study (Figure 2).

In the following, we will discuss our descriptive sensory analysis (sensory evaluation spectrum) to open up new possibilities for exploring or gaining a deeper understanding of humans. In the final study, we examined the relationship between mood change and odor and its physiological effects by focusing on the possible verbal and nonverbal changes in humans induced by smelling the fragrances of essential oils. Here an inquiry assessment was employed for evaluating changes in perception of a given aroma and these tests were conducted twice, when the subject was undergoing the Kraepelin mental performance test (mental arithmetic) or an auditory task (listening to environmental natural sounds), once before the task (pre-task) and once after the task (post-task). In seeking to identify possible physiological changes while inhaling the odorants, skin thermometer studies were conducted as a function of the task assigned to subjects. Related to the hypothesis that the elucidation of psychological and physiological reactions to odors might provide information regarding olfactory discrimination and responses in humans, Figure 8 was constructed to illustrate how this kind of paradigm might be useful for establishing the potency of essential oils and understanding the nature of olfactory discrimination and responses in humans. When observed as a function of task assigned to the subjects, the peppermint spectrum associated with the auditory task was shown to be the reverse to the spectrum recorded when participants were engaged in mental arithmetic. It was also shown that decreasing tendency in fingertip skin temperature was a mutual phenomenon in both conditions. Similar results were found for spearmint and linalool, with the exception of a lack of skin temperature change when subjects engaged in mental arithmetic and inhaled linalool.

Figure 8 shows the finer nuances of how essential oils manifest their potency. When viewed through two-way “task windows” set up for subjects, it appeared that essential oils could be classified into roughly two groups: those with an upward tendency of fingertip skin temperature and those with a downward tendency, although some degree of divergence was observed in both (verbal and non- verbal) responses to odorants. Reaction to spearmint and linalool could be regarded as a sedative response because these showed a downward tendency in fingertip skin temperature. A significant difference with decreasing tendency in fingertip skin temperature was observed in the case of spearmint vs. doing mental arithmetic (at *p* < 0.01) as well as linalool vs. listening to environmental (natural) sounds (at *p* < 0.05).

Emotional stress is known to induce a slight increase in skin temperature during feelings of excitement or apprehension [33-37]. Although the process by which skin temperature undergoes changes due to emotional stress has been demonstrated by several authors, the mechanism by which skin temperature changes occur along with inhalation of odorous molecules remains unresolved, but some will be mentioned briefly. Based on findings that blood vessels are supplied only with vasoconstrictor efferents, it has been suggested that emotional stress leads to cutaneous vasoconstriction, thus lowering skin temperature [38-43]. The participation of sympathetic nerve-mediated vasodilation has also been reported [44-46], although there are no known vasodilator nerve fibers connected to the cutaneous vessels in humans [47]. Other details can be found in Sugawara *et al.* [14].

The results obtained also suggest that the administration of fragrances of peppermint, spearmint and linalool might cause a different subjective perception and a different odor response depending on the work assigned to subjects, given that the fragrance given to the subjects was the same. Indeed, Figure 8 shows subtle nuances related to how olfactory discrimination and responses take place in humans. This reminds us that odors act as neurophysiological stimuli by causing different perceptions and lead in turn to diverse odor reactions depending on the internal and extraneous conditions of the subjects, as reported by Lorig and Schwartz [48].

It is interesting to consider the current state of knowledge about olfaction [49-53]: (1) Approximately 1,000 different odorant receptors work in hair-like cilia of the olfactory neuron where the initial detection of odors takes place in the olfactory epithelium at the posterior of the nose. (2) The number of genes for coding odorant receptors is considered to be up to 1% of the mammalian DNA, making this the largest gene family thus far identified. (3) There are a vast number and a great variety of odorant receptors at the entrance to the olfactory system enabling discrimination of a large range of odors and subtle differences in odorant molecules. In each experimental run, as shown in Figure 8, the fragrant compound given to subjects was the same. Thus a similar set of odorant receptors were activated in each participant at the entrance to the olfactory system. Therefore, the subtle nuances of expression of odor discrimination and responses, as shown in Figure 8, can be considered to result from diversity upstream from this point in the process of olfaction.

Such an assumption might be of greater importance than generally recognized thus far. Humans can detect and discriminate a vast number of odors and even slight alterations in the structure of an odorous molecule. The number of odors we can reliably distinguish is estimated to be more than 10,000 [54,55]. As for enantiomeric pairs of odorants, which possess the same molecular structures except for the chiral portion, it is said that these can lead to profound changes in perceived odor quality. For instance, it has been shown that (+)-carvone is characterized as a caraway-like scent, while (-)-carvone is a spearmint-like herbal odor [56,57]. The enantiomers of carvotanacetone and trans-dehydrocarvone, both synthesized from (+)-carvone, are caraway-like; those from (-)-carvone are spearmint-like [58], and (+)- and (-)-linalool are petitgrain-like and lavender-like, respectively [59].

**Figure 9.**
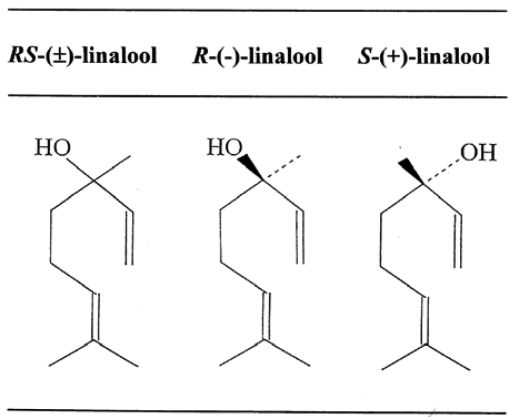
Formulas of the enantiomeric isomers of linalool.

It is of interest to refer to our previous findings of the sensory features of the enantiomeric isomers of linalool. Formulas of the enantiomers are shown in Figure 9. As described elsewhere [9,10], the linalool refined from lavender oil by repeated flash column chromatography was identified as (*R*)-(-)-linalool with an authentic (*R*)-form with a 97.0% purity on gas-liquid chromatography (GLC) with a specific rotation of [α]_D_ = -15.1. The enantiomer from coriander oil was identified as (*S*)-(+)-linalool with a purity of (*S*)-form 88.3% and (*R*)-form 11.7% on GLC and [α]_D_ = +17.4, while (*RS*)-(±)-linalool prepared from commercial linalool was identified as a racemic mixture of (*R*)-form (50.9%) and (*S*)-form (49.1%) with [α]_D_ = 0. Inhalation of (*R*)-(-)-linalool associated with listening to environmental (natural) sounds was found to produce a favorable impression in the sensory (verbal) tests, and this feature was quite similar to the respective spectrum of (*RS*)-(±)-linalool but not to that of (*S*)-(+)-linalool. On the other hand, administration of (*R*)-(-)-linalool associated with mental arithmetic was shown to be an unfavorable sensory feature, and this had a close resemblance to that of (*S*)-(+)-linalool as well as that of (*RS*)-(±)-l linalool even when undertaking mental arithmetic. It is worth noting the obtained values of a total significance score: 7.0 for (*R*)-(-)-linalool, 4.0 for (*S*)-(+)-linalool and 6.5 for (*RS*)-(±)-linalool when undertaking the auditory task; 4.5 for (*R*)-(-)-linalool, 3.5 for (*S*)-(+)-linalool and 4.0 for (*RS*)-(±)-linalool when undertaking mental arithmetic. These values demonstrate that the enantiometric isomers of linalool could be regarded as significantly different odors with dependence not only on species of enantiomer but also on tasks assigned to the subjects.

A great deal is already understood about the finer details of the unique features at the entrance of the olfactory system [49-53,60,61], but very little detail is known about processes upstream. These results indicate that further work is needed to characterize the framework for the discrimination of odorous molecules beyond the entrance to the olfactory system. Such information may provide clues to the following long-standing issues. How does the brain ultimately identify more than 10,000 odorants? How does the perception of odors usually associate with pleasant or unpleasant emotions? How does the brain prompt the range of emotional or behavioral responses that smells often provoke? How much of behavior or mood is governed by the perception of odors?

## 6. Conclusions

1. This paper is an overview of our verbal (semantic) research achievements over the past decade, suggesting that human senses might be indispensable sensors not only for practical uses but also for gaining a deeper understanding of humans. From this point of view, the following studies deserve emphasis.
2. In the first study, we used semantic responses from participants to determine the efficacy of the photocatalytic elimination of stains or bio-aerosols from an air environment using TiO_2_. Participants' impressions were recorded on an 11-point scale using 13 contrasting pairs of adjectives. Such an inquiry assessment was conducted twice: once for the TiO_2_ coated door and once for the uncoated door. The mean of the impression difference between the score of the first inquiry for the TiO_2_ coated door and the second inquiry for the uncoated door was plotted against the descriptors. The obtained bar graph (sensory evaluation spectrum) showed an upward tendency with a positive value against the descriptors. This suggests that the coated door was superior to the uncoated one related to the 13 setting semantic variables.
3. When a TiO_2_-type deodorizer was setting in the participants' own home refrigerators, in the second study, it was shown that a pair of sensory spectra could represent a functional aspect of the deodorizing efficacy of a photocatalytic deodorant: one was a spectrum for installation of deodorant in a refrigerator, and the other was for its removal.
4. In an attempt to shed light on the relationship between mood change and odor and its physiological effects in humans induced by smelling the fragrances of essential oils, in the final study, we focused on the possible verbal and nonverbal changes of the subjects while inhaling the fragrance of peppermint and spearmint essential oils and linalool. An inquiry assessment was employed for evaluating changes in perception of a given aroma, when the subject was undergoing the Kraepelin mental performance test (mental arithmetic) or an auditory task (listening to environmental natural sounds), once before the task (pre-task) and once after the task (post-task). To identify further possible physiological changes of inhaling the odorants, skin thermometer studies were conducted. The obtained findings give support to the conclusion, as reported by Lorig and Schwartz [48], that odors act as neurophysiological stimuli by causing different perceptions and lead in turn to diverse odor reactions depending on the internal and extraneous conditions of the subjects.

## Figures and Tables

**Table 1.** Twenty-three contrasting pairs of adjectives selected by five panelists for assessing the efficacy of photocatalytic elimination of stains or bio-aerosols from air environments by TiO_2_.

bright – dark	neat – sluggish
clear – heavy	pure – sandy
clean – dirty	natural – artificial
calm – irritating	harmonious – inharmonious
agreeable – disagreeable	comfortable – uncomfortable
refined – vulgar	likeable – dislikable
fresh – stale	fine – dusty
transparent – opaque	airy – murky
dry – watery	brilliant – stringy
smooth – rough	glossy – gloomy
slippery – coarse	light – sticky
simple – thick	
